# Quality and Functional Breadth of Publicly Available Mobile Apps Supporting Self-Management After Kidney Transplantation: Cross-Sectional App Store Evaluation

**DOI:** 10.2196/92472

**Published:** 2026-09-30

**Authors:** Hyejin Lee, Chan Mi Kang

**Affiliations:** 1 Department of Nursing Research Institute of Dong-Eui Nursing Science Dong-Eui University Busan Republic of Korea; 2 Department of Nursing Division of Health Science Dongseo University Busan Republic of Korea

**Keywords:** kidney transplantation, self-management, mobile apps, mobile health, mhealth, app quality, mobile app rating scale

## Abstract

**Background:**

Nonadherence to immunosuppressants and other self-management behaviors remains a major challenge after kidney transplantation. Publicly available mobile apps may support long-term self-management, but their quality and functional breadth remain unclear.

**Objective:**

This study aimed to identify publicly available mobile apps accessible in South Korea that support self-management after kidney transplantation, evaluate their quality and functional breadth using the Mobile App Rating Scale (MARS) and the IMS Institute for Healthcare Informatics functionality score, and describe gaps in transplant-specific self-management support.

**Methods:**

From January 13 to 21, 2026, we searched the Apple App Store and the Google Play Store using 6 keywords, including “kidney transplant” and “kidney graft,” and selected apps that met the inclusion criteria (n=6). Two trained nursing faculty raters independently evaluated each app using the MARS and IMS functionality score. Interrater reliability was good for the MARS (intraclass correlation coefficient=0.71, 95% CI 0.62-0.78) and the IMS functionality score (Cohen κ=0.88).

**Results:**

The mean MARS score across the 6 apps was 4.06 (SD 0.96). Subscale scores were highest for aesthetics (mean 4.22, SD 0.81), followed by functionality (mean 4.13, SD 0.80), information (mean 4.11, SD 0.75), and engagement (mean 3.80, SD 1.32). The mean IMS functionality score was 5.67 (SD 4.27) out of 11 functions, and the “evaluate data” function was included in only 1 (16.7%) app.

**Conclusions:**

Few publicly available apps met the eligibility criteria. Although trained-rater MARS assessments were generally favorable, functions involving data evaluation and individualized guidance or intervention were less frequently implemented. Because the apps differed in their intended purposes and the study did not assess end-user usability or clinical outcomes, the findings should be interpreted as a descriptive baseline rather than evidence of usability or effectiveness. Future studies should involve kidney transplant recipients in patient-centered usability and outcome evaluations.

## Introduction

### Self-Management After Kidney Transplantation

Kidney transplantation is considered the optimal treatment for patients with end-stage renal disease and is actively performed worldwide. Of approximately 173,000 organ transplants performed globally in 2024, kidney transplantation accounted for the largest proportion [1]. In the United States, 28,142 individuals underwent kidney transplantation in 2023 [2], and in South Korea, 1704 individuals underwent kidney transplantation in 2024 [3], demonstrating that kidney transplantation is widely implemented as a key treatment modality in many countries. However, even after a successful transplant, recipients remain at high risk over time for health problems related to immunosuppressive therapy, including infections, increased malignancy, hypertension, diabetes mellitus, dyslipidemia, cardiovascular disease, reduced muscle strength, and osteoporosis [4-6]. To prevent these complications and maintain graft function, comprehensive self-management is essential, including adherence to immunosuppressant regimens, early detection of rejection, prevention of complications and infections, and management of diet and daily life [7].

Although inpatient education and regular outpatient counseling have traditionally been provided to support self-management among kidney transplant recipients, these conventional interventions have limitations for continuous and comprehensive monitoring. Such limitations include the restricted duration of hospitalization, long intervals between outpatient visits (typically every 3 months), and difficulties in accurately assessing patients’ actual self-management behaviors in real-world settings [7]. In fact, the nonadherence rate to immunosuppressive medication—the most critical aspect of posttransplant care—increases sharply and continues to worsen, rising from 11% in the early period to 31% at 3 months after transplantation [8]. Nonadherence to dietary management is also substantial; adherence rates are only 15% for a low-sodium diet and 8% for a low-fat diet [9]. Nonadherence rates for self-monitoring of vital signs and rejection surveillance exceed 90% [10]. Moreover, as time elapses after transplantation, adherence to immunosuppressive medication decreases by 4.8% annually [11]. The economic burden associated with such nonadherence is considerable; compared with adherent patients, nonadherent patients incur an additional US $100,000 to US $300,000 in cumulative medical costs over a 3-year period [12].

### Mobile Apps for Posttransplant Self-Management

Nonadherence to self-management among kidney transplant recipients arises from a range of factors, including forgetfulness, the complexity of multidrug regimens, and fear of graft failure [13]. Accordingly, multifaceted support—such as medication reminders, education, and real-time monitoring—is essential [14]. Mobile apps have been used as an intervention modality capable of providing such support, and a recent meta-analysis involving kidney transplant recipients reported significant beneficial effects of mobile app–based interventions [15]. When mobile apps are used to support self-management after kidney transplantation, they can improve medication adherence more rapidly than conventional face-to-face interventions and promote sustained self-management. In addition, automated medication reminders, real-time feedback, and continuous monitoring can enhance patients’ self-efficacy and facilitate the long-term maintenance of self-management behaviors [16].

Although approximately 15 to 20 clinical studies on mobile apps for self-management in kidney transplant recipients have been reported in the scientific literature [17], only a limited number of apps are actually available for download in commercial app stores, such as Kidney Transplant Compare and Kidney Transplant Education. Most apps remain at the clinical trial stage or are provided only within specific health care institutions. In South Korea, a mobile app to support postoperative self-management for kidney transplant recipients was developed in 2016 [18] and was judged to have high content validity by experts; however, it was not commercialized. As a result, kidney transplant recipients currently have limited access to effective self-management mobile apps in real-world settings. Therefore, to overcome these domestic limitations, it is necessary to examine the current status of kidney transplant self-management apps developed and used internationally and to systematically analyze the functions and quality of each app. Such analysis can inform the design principles and core functional components that should be considered when developing apps in South Korea.

### Study Objective

This study aimed to identify publicly available mobile apps accessible in South Korea that support self-management after kidney transplantation, evaluate their quality and functional breadth using the Mobile App Rating Scale (MARS) and the IMS Institute for Healthcare Informatics functionality score, and describe gaps in transplant-specific self-management support.

## Methods

This cross-sectional descriptive app store evaluation identified publicly available mobile apps accessible in South Korea that supported self-management after kidney transplantation and assessed their quality and functional breadth. Relevant elements of the PRISMA (Preferred Reporting Items for Systematic Reviews and Meta-Analyses) 2020 framework were adapted to enhance the transparency and reproducibility of the app search and selection process.

### App Search and Eligibility

From January 13 to 21, 2026, we searched and analyzed mobile apps designed to support self-management for kidney transplant recipients in South Korea’s 2 major app stores: the Apple App Store and Google Play Store. The search was intentionally limited to these 2 publicly accessible consumer app stores because the study focused on apps that users in South Korea could independently discover and download through standard public channels; institution-specific and access-restricted apps were outside the predefined study scope. Two reviewers (HL and CMK) independently searched both platforms using 6 keywords: “신장이식,” “이식,” “kidney transplant,” “kidney graft,” “renal transplant,” and “renal graft.” Apps were eligible if they (1) were available in Korean or English and (2) included functions that directly supported self-management among kidney transplant recipients (eg, medication reminders, health monitoring, dietary management, and symptom tracking). We excluded duplicate apps released in different versions (eg, “Pro” and “Lite”), apps requiring restricted registration or an access code, and apps that were not available or did not function in South Korea. Discrepancies were resolved through consensus to determine the final set of apps for analysis.

### App Quality and Functional Measures

#### Mobile App Quality Assessment Tool

The MARS is an instrument developed by Stoyanov et al [19] to assess the quality of mobile apps. It consists of 29 items encompassing 4 objective quality domains and 2 subjective quality domains. The objective domains include engagement, functionality, aesthetics, and information. The engagement domain comprises 5 items assessing entertainment, interest, customization, interactivity, and content appropriateness. The functionality domain includes 4 items evaluating app performance (accuracy and speed), ease of use, navigation, and gestural design and consistency. The aesthetics domain includes 3 items addressing layout, graphics quality, and visual appeal. The information domain comprises 7 items assessing the accuracy of the app description, goals, quality of information, quantity of information, visual presentation of information, credibility, and evidence base. The MARS also includes a Subjective Quality section and an App-Specific section. The Subjective Quality section comprises 4 items assessing willingness to recommend the app, intended frequency of use (over the next year), willingness to pay, and overall star rating. The App-Specific section comprises six additional items assessing the perceived impact of the app on awareness, knowledge, attitudes, motivation, help-seeking, and behavior change. Each item is rated on a 5-point scale ranging from 1 (“inadequate”) to 5 (“excellent”), with higher scores indicating higher app quality. In the original development study, MARS demonstrated high internal consistency (Cronbach α=0.90) and good interrater reliability (intraclass correlation coefficient [ICC]=0.79). In the present study, the MARS demonstrated good internal consistency (Cronbach α=0.84) and good interrater reliability (ICC=0.71, 95% CI 0.62-0.78).

#### Mobile App Functionality Analysis Tool

The IMS functionality score is a tool developed based on a report from the IMS Institute for Healthcare Informatics to analyze the functions of mobile apps [20]. It assesses 11 items across 7 functional domains: inform (provide information), instruct (provide instructions), record, display, guide, remind or alert, and communicate. Within the record domain, data functions are further categorized into 4 subcomponents: collect data, share data, evaluate data, and intervene. Each item is scored as 1 if the function is present and 0 if absent; higher total scores indicate that an app incorporates a broader range of functions. Although no prior studies have formally reported the reliability of the IMS functionality score, it has been used in multiple studies to analyze app functionality [21-25]. In the present study, interrater reliability for the IMS functionality score was high (Cohen κ=0.88; *P*<.001).

### Rater Qualification and Training

Two raters (HL and CMK), both of whom were nursing faculty members, conducted the app evaluations. Both raters had research expertise in self-management after kidney transplantation and clinical experience caring for kidney transplant recipients and patients with chronic conditions. They also had prior experience in mobile health research and in evaluating mobile health apps using the MARS. Before the formal evaluation, the raters independently assessed 3 apps and compared their ratings to clarify the interpretation of individual MARS items and response categories. Any discrepancies were discussed with reference to the original MARS definitions and scoring guidelines to establish consistent evaluation criteria. Because this study was designed as a trained-rater assessment rather than an end-user usability evaluation, the MARS was considered appropriate for the study purpose.

### Evaluation Procedures

This study was conducted after obtaining an exemption from institutional review board review (2025-025-HR-01). For the main evaluation, both raters independently assessed all included apps using the MARS and IMS functionality score. Ratings were completed independently before interrater reliability was calculated. Afterward, discrepancies were discussed, and final consensus ratings were determined with reference to the original scoring guidelines.

### Data Analysis

Scores obtained using the MARS and IMS functionality score were summarized as means and SDs using Microsoft Excel. Interrater reliability was assessed using the 2 raters’ independent ratings obtained before consensus and analyzed with SPSS Statistics (version 29.0; IBM Corp). For the MARS, which was treated as a continuous measure, a 2-way random-effects, absolute-agreement, single-measure ICC was calculated. For the IMS functionality score, which consisted of dichotomous item ratings (0=absent, 1=present), unweighted Cohen κ was calculated. ICC and Cohen κ values of 0.60 or higher were considered acceptable.

## Results

### Selection of Apps for Analysis

In this study, a total of 146 kidney transplant–related apps were identified across the iOS and Android platforms using the predefined keywords. First, 27 (18.5%) duplicate apps were removed by checking app titles and developer names (n=119, 81.5%). Next, app titles, descriptions, and screenshots were reviewed in detail; 94 (79.0%) apps unrelated to kidney transplant self-management and 2 (1.7%) apps available only in languages other than Korean or English were excluded (n=23, 19.3%). After downloading the remaining 23 (19.3%) apps, additional exclusions were applied for apps that did not directly support self-management for kidney transplant recipients, apps not accessible in Korea, and apps restricted to study participants only. Ultimately, 6 (4.1%) apps intended to support self-management among kidney transplant recipients were included in the final analysis (Figure 1).

**Figure 1:**
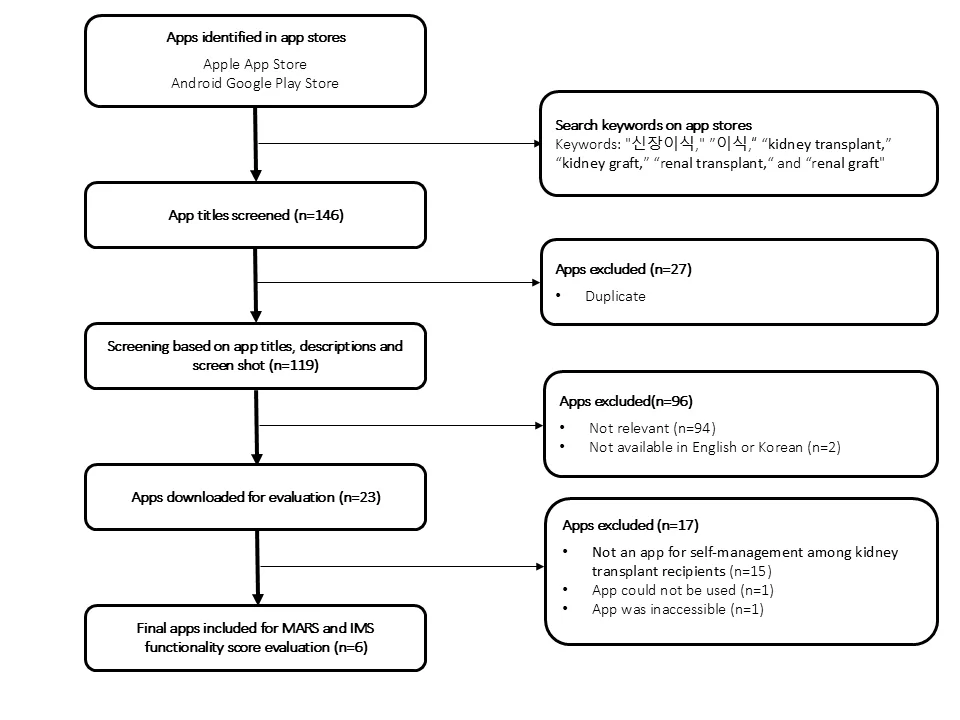
App selection process flowchart. MARS: Mobile App Rating Scale.

### Descriptive Characteristics

The general characteristics of the 6 apps are presented in Table 1. Apps available for download on only 1 platform (iOS or Android) accounted for 66.7% (n=4), whereas 33.3% (n=2) were available on both platforms. Among the 5 apps for which the last update date was available, 4 (80%) had been updated since 2025, whereas 1 (20%) was last updated in 2020; the update date was not available for 1 app. All apps were free to download. The number of downloads on Android varied (ie, ≥1000 to <10,000 or ≥10,000), whereas download counts could not be confirmed for iOS. After installation, 66.7% (n=4) could be used immediately without a separate login process, while 33.3% (n=2) required account creation and login.

**Table 1:** Description of included apps.

Name	Developer	Developer type	Platform	Version	Last updated	Cost (US $)^a^	Downloads	Login	Language
Mizu-Your CKD companion	Carealytix Digital Health GmbH	Company	iOS and Android	3.12.0	2025.12.11	Free	>10,000	Yes	English
Organ Transplant Center Medication Reminder	Samsung Medical Center	Hospital	Android	1.1.12	2025.8.25	Free	>1000	No	Korean
AlloCare Transplant Health	CareDx	Company	iOS	5.1.3	2025.4.9	Free	—^b^	Yes	English and Spanish
Kidney Transplant TXP	The University of Michigan	University	iOS and Android	1.4.1	2025.10.29	Free	>1000	No	English
TransplantU	University of Nebraska Medical Center	University	iOS	—	NA	Free	—	No	English
Kidney Transplant	Phoenix Children’s Hospital Inc	Company	iOS	1.1	2020.2.14	Free	—	No	English

^a^In-app cost and download cost.

^b^Not available.

### MARS and IMS Functionality Scores

#### MARS Quality Scores

Using MARS, the 6 kidney transplant self-management apps had a mean objective quality score of 4.06 (SD 0.96) across engagement, functionality, aesthetics, and information. Three (50%) apps scored ≥4.0/5 (Table 2 and Multimedia Appendix 1). Aesthetics rated highest, followed by functionality, information, and engagement. Subjective quality scores were slightly lower than objective scores.

The aesthetics domain (layout, graphics, and visual appeal) had a mean score of 4.22 (SD 0.81). In 66.7% (n=4) of the apps, images and icons were rated as appropriately arranged, and the main-screen design and graphic quality were rated highly. No app received an aesthetics score below 3.

The functionality domain (performance, ease of use, navigation, and gestural design) had a mean score of 4.13 (SD 0.80). Of the 6 apps, 4 (66.7%) could be used without account creation or login. In addition, 2 (33.3%) apps provided user instructions. These instructions were presented as text, images, videos, or pop-up messages, and were accompanied by in-app icons and corresponding labels.

The information domain (accuracy of description, goals, information quality and quantity, visual information, credibility, and evidence base) averaged 4.11 (SD 0.75). App descriptions were rated as appropriate in 5 (83.3%) apps, and the stated goals of all 6 apps were rated as clear and appropriate. Information quality was generally rated favorably across the apps; however, none had an evidence base demonstrating clinical effectiveness.

The engagement domain (entertainment, interest, customization, interactivity, and target group) had a mean score of 3.80 (SD 1.32). In 4 (66.7%) apps, entertainment value and perceived interest were rated as high. Mizu-Your CKD Companion enhanced user interest by incorporating a familiar raccoon character and presenting content using graphics. In TransplantU, education was delivered through interaction with a customizable character, and additional features (eg, attaching or removing equipment on the character) were included to increase engagement. Personalization options (eg, sound, content, and alarms) were available in only 50% (n=3) of the apps, while 66.7% (n=4) supported data sharing and feedback functions.

Based on the objective MARS scores, the highest-scoring apps were Mizu-Your CKD Companion (4.85), Organ Transplant Center Medication Reminder (4.49), and AlloCare Transplant Health (4.08).

**Table 2:** Mobile App Rating Scale (MARS) and IMS functionality scores.

Name	MARS score (app quality ratings)						MARS score (subjective quality)		MARS score (app-specific)		IMS score
	Engagement	Functionality	Aesthetics	Information	Overall, mean (SD)						
Mizu-Your CKD companion	5.00	4.75	5.00	4.67	4.85 (0.38)	4.75		5.00		11	
Organ Transplant Center Medication Reminder	4.20	4.75	4.33	4.67	4.49 (0.51)	4.00		4.00		7	
AlloCare Transplant Health	4.40	3.25	4.67	4.00	4.08 (0.73)	3.00		3.50		10	
Kidney Transplant TXP	3.00	4.25	4.00	4.50	3.94 (1.06)	3.50		3.50		2	
TransplantU	3.80	3.50	4.00	3.50	3.70 (1.03)	3.00		3.00		1	
Kidney Transplant	2.40	4.25	3.33	3.33	3.33 (1.02)	1.75		2.50		3	
Score, mean (SD)	3.80 (1.32)	4.13 (0.80)	4.22 (0.81)	4.11 (0.75)	4.06 (0.96)	3.33 (1.13)		3.58 (1.05)		5.67 (4.27)	

#### IMS Functionality Scores

The results of the IMS functionality assessment are presented in Table 2, Figure 2, and the Multimedia Appendix 1. Across the 6 apps, the mean number of functions implemented per app was 5.67 (SD 4.27). By function, inform was the most frequently implemented (n=5, 83.3%), followed by instruct, record, and communicate (each n=4, 66.7%). Collect data, share data, display, and remind or alert were each present in 50% (n=3) of the apps, while intervene and guide were each observed in 33.3% (n=2). Evaluate data was the least frequently implemented function, identified in only 16.7% (n=1) of the apps. At the app level, Mizu-Your CKD Companion included all 11 functions, and AlloCare Transplant Health implemented 10 functions, excluding evaluate data.

Across the 6 kidney transplant self-management apps, inform was present in 83.3% (n=5) and provided posttransplant information (medication, daily life, diet, and required tests) via videos, YouTube, and websites. Instruct was present in 66.7% (n=4) and offered app-use guides and instructions for interpreting in-app tables and charts. Record was present in 66.7% (n=4) and allowed entry of laboratory results, vital signs (temperature, blood pressure, and pulse), and physical and biometric measures (height, weight, and blood glucose). Data-related functions were analyzed as collect data, share data, evaluate data, and intervene. Collect data was present in 50% (n=3) and stored user-entered data. Share data was present in 50% (n=3) and enabled sharing via email or text messages. Evaluate data was present in 16.7% (n=1) and analyzed data to identify self-management patterns or assess goal attainment. Intervene was present in 33.3% (n=2) and provided medication-time alerts and coping strategies with alerts when abnormal data were entered. Display was present in 50% (n=3) and showed test results and vital signs as graphs or images. Guide was present in 33.3% (n=2) and provided behavioral guidance based on user input. Remind or alert was present in 50% (n=3) and prompted users to enter self-management behaviors. Communicate was present in 66.7% (n=4) and supported communication with other users or clinicians via boards or email.

**Figure 2:**
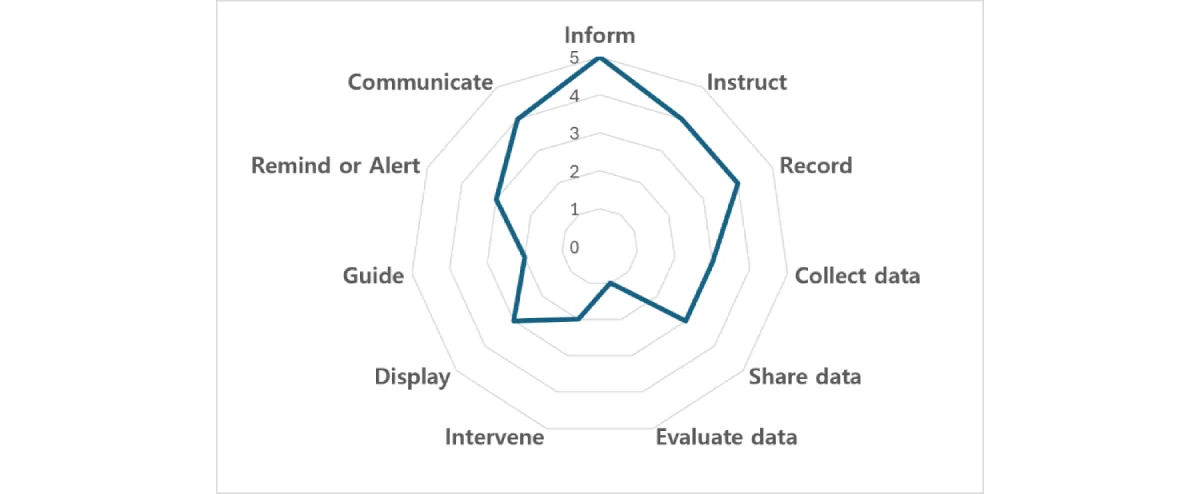
Number of apps implementing each IMS functionality (n=6).

### Description of the Top 3 Highest-Scoring Apps

The 3 apps with the highest objective MARS scores were selected for further descriptive comparison, and their IMS functionality scores were also considered. Key features of the selected apps were presented as screenshots (Figure 3).

**Figure 3:**
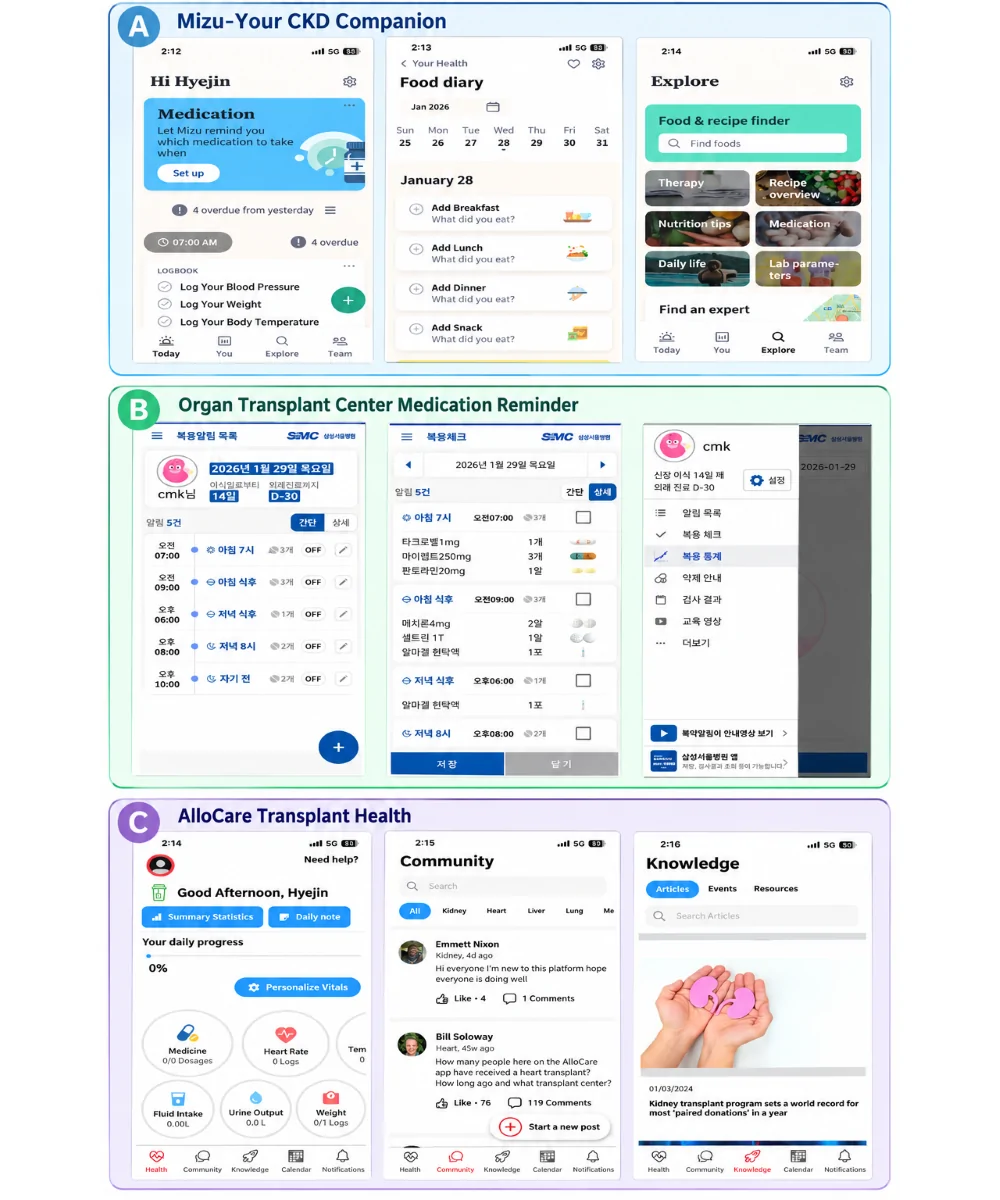
Screenshots of the top 3 apps.

#### Mizu-Your CKD Companion

This mobile app was developed by Carealytix Digital Health GmbH (Germany) to support comprehensive self-management for patients with chronic kidney disease (CKD). It was designed to cover all stages of kidney disease, from early stage to dialysis and posttransplant care. In this study, it was judged to provide posttransplant management functions and was therefore included in the final selection. Its core features include a vital-parameters logbook, kidney-specific food diary, medication tracking, weekly routine customization, educational content, a travel dialysis finder, and a community and support network. Transplant-specific functions include kidney function assessment for graft health monitoring, fluid balance and blood pressure management, early detection of graft rejection, infection monitoring, immunosuppressant management and medication monitoring, and management of posttransplant diabetes and metabolic complications.

The app achieved the highest MARS score among the top 3 apps (4.85) and received perfect scores (5.0) in the engagement and aesthetics domains. In the engagement domain, entered health parameters are displayed as real-time graphs, enabling immediate review of trends. Tracking items are automatically configured based on the user’s kidney function status (estimated glomerular filtration rate [eGFR]). Users can customize weekly routine items and logging frequency, and the app supports interactive functions such as medication reminders. Development involved collaboration with nephrologists and university hospitals, and the user interface is designed to be intuitive for older adults. In the aesthetics domain, complex functions are organized within a clear hierarchical structure, with appropriate button and input font sizes. High-resolution icons and a traffic-light system (green: within target range; yellow: caution; red: risk) facilitate rapid recognition of health status and prompt user action. However, because no study has directly evaluated clinical effectiveness, the evidence base item in the information domain could not be scored. The initial setup process (eg, age, sex, height, weight, and CKD stage) is relatively complex.

The IMS functionality score was 11 out of 11, indicating that the app incorporated all functions. It provides kidney disease–related information in multiple formats (text, images, and videos) and includes tutorials and function-specific tips to support correct use. Users can record health parameters (eg, blood pressure and blood glucose), diet, and medication adherence and can share data with clinicians by generating PDF reports and exporting via email. Complex health data are visualized using various charts, and tailored feedback is delivered based on user-entered data. The app includes medication reminders and alerts for abnormal biometric values, and users can email their information to clinicians prior to clinic visits.

#### Organ Transplant Center Medication Reminder

This Korea-based app was developed in 2018 to support accurate medication adherence after solid-organ transplantation and to provide transplant-related information. Core functions include medication reminders, adherence statistics, entry and trend tracking of key laboratory test results, medication information (including immunosuppressants), and transplant-related educational videos. The app has been downloaded more than 1000 times to date and is available only in Korean.

The app received an overall MARS score of 4.49, with near-ceiling scores in the functionality and information domains. In the functionality domain, the app demonstrated excellent performance, with no technical errors in in-app features or in button and menu operation and no loading delays. An instructional video is provided to facilitate user uptake. Navigation was logical, easy, clear, and intuitive, enabling a smooth screen flow throughout (“perfectly logical, easy, clear and intuitive screen flow throughout”). In the information domain, the app presented clearly specified goals that were measurable and achievable. The quality of information—covering medication information, post–kidney transplant lifestyle guidance, immediate posttransplant management priorities, recovery-phase care, and long-term management—was highly relevant, appropriate, coherent, and correct. The quantity of information was comprehensive yet concise, and the app included links to additional information and resources. Most content was delivered via videos with clear, logical, and accurate visualization. However, information sources were not cited, and the app lacked evidence from effectiveness and validation studies, which lowered its score in the evidence base component. In the aesthetics domain, the layout was professional, simple, clear, and orderly, with device-optimized display and purposefully arranged design components. Finally, the engagement domain averaged 4.2. Users could customize alarm sounds; however, this personalization was limited to medication reminders. Although the app allowed entry of medication adherence, blood pressure, weight, urine output, and laboratory values (blood urea nitrogen and creatinine), feedback was restricted to medication adherence rates. In addition, while posttransplant management information and frequently asked questions were provided, the app did not include functions for recording dietary or exercise management or receiving feedback on these behaviors.

The app received an IMS functionality score of 7, indicating that user-entered data could be collected, displayed (graphically), and shared. Data entry primarily focused on medication adherence and key laboratory test results, which were presented in graph format. However, several limitations were identified. The app did not include functions to evaluate collected data, provide tailored interventions based on these evaluations, or recommend clinician consultation when needed. In addition, it lacked data entry functions for exercise and dietary management.

#### AlloCare Transplant Health

This app was developed in 2022 to support day-to-day health management after solid-organ transplantation and to facilitate communication with others who have similar lived experiences. Key services include wellness activity tracking, enabling users to track medication, fluid intake, urine output, heart rate, blood pressure, steps, weight, temperature, blood sugar, mood, and sleep. Through the transplant community feature, users can share experiences and engage in discussions with peers. The app also provides a range of transplant-related articles and videos. In addition, the Labs function, including AlloSure, allows users to transmit laboratory results to physicians to support monitoring of graft function. Use of the app requires login, and it is available in Spanish and English.

The app received an overall MARS score of 4.08, with aesthetics rated highest, followed by engagement, information, and functionality. In the aesthetics domain, the size and placement of on-screen buttons, icons, and menus were mostly clear. The graphics and visual design applied to buttons, icons, menus, and content were high in quality and resolution and stylistically consistent. Overall, the app was perceived as visually very attractive. In the engagement domain, app use was moderately fun and entertaining. The provision of diverse articles and events, together with communication with peers who had similar experiences, was considered sufficient to sustain interest and encourage repeated use relative to other apps. Medication reminders allowed flexible scheduling, and users could customize biometric reminders as well as community and knowledge notifications. Notably, users could reorder the display sequence of biometric data to enable rapid access to prioritized information. During medication and biometric data entry, the app offered multiple interactive elements, including reminders, sharing functions, and notifications. In the information domain, the app included functions to support daily self-management after kidney transplantation by enabling tracking of medication, diet, health status, sleep, and mood. In addition, the inclusion of social communication features to mitigate burden and fatigue related to self-management suggested a high likelihood of achieving its intended goals. In the functionality domain, performance limitations were observed: some contents in the knowledge menu intermittently failed to load, and the app occasionally slowed. Given the breadth of functions, initial learning required some time and effort.

The app demonstrated a relatively broad feature set (IMS functionality score of 10). It provided transplant-related information via text, images, and videos and enabled saving, collecting, and sharing of user-entered data. The app also supported intervention functions, including behavioral guidance based on user inputs and warning and alert notifications, and allowed communication with health care professionals and/or other patients. However, it did not support shared evaluation of entered health data among stakeholders (eg, between patients and health care professionals, health care professionals and administrators, or patients and caregivers).

## Discussion

This study identified mobile apps designed to support self-management in kidney transplant recipients and evaluated their quality, functionality, and content to inform future app development. Prior reviews examined self-management apps for patients undergoing dialysis [26-28] and smartphone apps for CKD [29], but kidney transplant self-management apps have not been evaluated.

### Principal Findings

Across the 6 apps analyzed, all included educational functions that provided information to improve users’ physical health and disease-related knowledge. In addition, half of the apps (n=3, 50%) monitored and tracked user-entered data to promote behavior change and improve quality of life, and 2 (33%) apps provided tailored advice and tips based on the recorded information. These apps supported behavior change by sending medication reminders and visually displaying daily lifestyle patterns (eg, diet and exercise).

The 6 kidney transplant self-management apps had a mean MARS score of 4.06 (SD 0.96), indicating that most were of sufficient quality for potential clinical use, with relatively high ratings for esthetics and functionality. However, the IMS functionality analysis showed that the apps primarily focused on inform and record, whereas evaluate functions that clinically interpret user-entered data were rare (n=1, 16.7%), and guide and intervene functions that deliver individualized behavioral strategies were limited (each n=2, 33.3%). These findings suggest that even when apps are visually appealing and easy to use, the core self-management cycle—data evaluation, feedback, and intervention—may not be adequately implemented. Overall, the apps performed relatively well in collecting, displaying, and sharing data, whereas functions for evaluating collected information and translating it into actionable next steps were less frequently implemented. However, the apps varied substantially in their intended purposes. Some were designed as focused medication adherence tools, whereas others aimed to provide broader kidney or transplant self-management support. Accordingly, a lower IMS functionality score should not necessarily be interpreted as poor performance when an app successfully fulfills its stated purpose, because the IMS functionality score primarily reflects functional breadth rather than effectiveness or purpose-specific quality. From the perspective of comprehensive kidney transplant self-management, however, the limited availability of data evaluation, individualized guidance, and intervention functions represents an important unmet need. This is particularly relevant in kidney transplantation, where self-management requires early recognition and timely responses to warning signs related to immunosuppressant adherence, infection symptoms, fluid balance, blood pressure and glucose control, and changes in graft function [7]. Nevertheless, most apps did not provide structured interventions such as risk-based alerts, threshold-triggered recommendations, or prompts to seek clinical consultation. This pattern is consistent with a review of genetic and genomic patient apps [30], which reported an average of only 3.3 of 11 functions, with 0% providing evaluate functions and engagement scoring the lowest.

Engagement differed across kidney transplant self-management apps primarily by the level of customization and interaction quality, rather than by visual appeal alone. Mizu supported rapid status recognition and behavior change through real-time graph updates, eGFR-based personalization, user-adjustable routines, and a traffic-light system. AlloCare promoted repeat use via community and content (events and articles), although menu errors and slow loading may impede sustained use. Medication Reminder provided reminder-based interactions, but feedback was largely limited to adherence, offering weaker support for lifestyle change (eg, diet and exercise). Overall, engagement may be enhanced by integrating structured behavior-change strategies, including personalized goals, immediate feedback, data-driven alerts, and reinforcement through achievement and/or social support.

The mean MARS information score was high (4.11, SD 0.75) and inform was the most frequently implemented IMS function (83.3%), confirming that posttransplant education on medication, daily life, diet, and testing is a central role of current apps. This is clinically relevant because kidney transplant recipients can revisit self-management information at any time after discharge. However, most apps lacked direct effectiveness evaluations or provided limited supporting evidence. MARS assesses the evidence base separately because, without demonstrated effects on clinical outcomes (eg, adherence, emergency department visits, hospitalization, graft function, and quality of life), clinicians cannot formally recommend or integrate an app into care [19]. Although prior studies have reported beneficial effects of mobile health–based self-management interventions in kidney transplant recipients [18,31], many commercial app store products have not undergone stepwise validation of development, usability, validity, and effectiveness. Rigorous studies are needed to establish the clinical effectiveness of commercial apps.

Effective kidney transplant self-management requires tailored apps that reflect transplant-specific needs from the design stage. Unlike patients with CKD, whose primary goal is to slow disease progression, kidney transplant recipients must maintain graft survival through lifelong, precise immunosuppressant adherence and early detection of rejection. The included apps varied in scope, with some targeting kidney transplantation specifically and others addressing broader kidney disease or solid-organ transplant populations. This heterogeneity suggests that the content and functions of broader kidney disease apps may not fully align with the distinct self-management needs of kidney transplant recipients.

These findings suggest areas for future formative research rather than prescriptive requirements for app design or clinical workflows. Future app development should involve kidney transplant recipients and health care professionals in co-design and usability testing and should examine whether transplant-specific monitoring, personalized feedback, and communication functions can be implemented safely and improve self-management or clinical outcomes. Specific alert thresholds, clinician response times, and clinical workflow requirements should be established through clinical consensus and prospective evaluation rather than inferred from the present app assessment.

### Limitations and Strengths

The limitations of this study are as follows. First, we evaluated only apps released in Korean or English and accessible or usable in South Korea. Consequently, apps released in other languages, those requiring access codes, those distributed exclusively within specific health care institutions, and newly released apps after the study period may not have been included. Second, because the MARS and IMS assess app status at a single time point, they cannot capture changes in usability with long-term use or the effects of subsequent feature updates. Our assessment reflects the apps as of January 2026; thus, the time lag to publication should be considered. Third, this study assessed app quality and functional breadth from the perspectives of 2 trained nursing faculty raters with relevant clinical and mobile health research experience. Although their expertise supported the structured application of the MARS and IMS functionality score, the study did not evaluate end-user usability, acceptability, satisfaction, sustained engagement, or clinical effectiveness among kidney transplant recipients. Therefore, the findings should not be interpreted as evidence that the evaluated apps are usable, acceptable, or clinically effective for kidney transplant recipients. Future studies should involve kidney transplant recipients in usability testing and co-design and should evaluate adherence, self-management behaviors, and clinical outcomes. Fourth, only a small number of eligible apps were identified, and the included apps varied in their target populations and intended purposes. This heterogeneity limited direct comparisons and the generalizability of the aggregate MARS and IMS findings.

Despite these limitations, this study provides a structured baseline description of publicly available apps accessible to kidney transplant recipients in South Korea. The combined use of the MARS and IMS functionality score provided complementary information on trained-rater quality assessments and functional breadth. These findings identify areas that can be examined in subsequent patient-centered co-design, usability, safety, and effectiveness studies.

### Conclusions

This cross-sectional app store evaluation identified 6 publicly available apps accessible in South Korea that supported at least one aspect of self-management after kidney transplantation. Trained-rater MARS assessments were generally favorable, whereas IMS analysis showed that data evaluation and individualized guidance or intervention functions were less frequently implemented. Because the apps differed in their intended purposes and no end-user usability or clinical outcomes were assessed, the findings should be interpreted as a descriptive baseline rather than evidence of usability or effectiveness. Future studies should involve kidney transplant recipients in co-design and patient-centered usability and outcome evaluations.

## Appendix

Multimedia Appendix 1 Mobile App Rating Scale (MARS) and IMS data.

## Abbreviations

CKD: chronic kidney disease
eGFR: estimated glomerular filtration rate
ICC: intraclass correlation coefficient
MARS: Mobile App Rating Scale
PRISMA: Preferred Reporting Items for Systematic Reviews and Meta-Analyses

## Data Availability

The app-level consensus ratings, Mobile App Rating Scale domain scores, and IMS item-level coding generated in this study are available from the corresponding author upon reasonable request. No personal or patient data were collected.
