# Supplementary material for: Quality and Functional Breadth of Publicly Available Mobile Apps Supporting Self-Management After Kidney Transplantation: Cross-Sectional App Store Evaluation
Source: JMIR Form Res. 2026 Sep 30;10:e92472. doi: 10.2196/92472 (PMC13669712; doi:10.2196/92472)
Supplement: Supplementary file 1 [file formative_v10i1e92472_app1.docx]

Appendix table

| App title  Tools | | Mizu-Your CKD companion | Organ Transplant Center Medication Reminder | AlloCare Transplant Health | Kidney Transplant TXP | TransplantU | Kidney Transplant |
| --- | --- | --- | --- | --- | --- | --- | --- |
| MARS | 1.Entertainment | 5 | 4 | 4 | 3 | 5 | 3 |
|  | 2.Interest | 5 | 4 | 5 | 3 | 4 | 3 |
|  | 3.Customisation | 5 | 4 | 4 | 1 | 1 | 1 |
|  | 4.Interactivity | 5 | 4 | 4 | 3 | 4 | 1 |
|  | 5.Target group | 5 | 5 | 5 | 5 | 5 | 4 |
|  | **Mean score** | 5 | 4.2 | 4.4 | 3 | 3.8 | 2.4 |
|  | 6.Performance | 5 | 5 | 3 | 4 | 5 | 4 |
|  | 7.Ease of use | 4 | 4 | 3 | 5 | 3 | 5 |
|  | 8.Navigation | 5 | 5 | 3 | 4 | 3 | 4 |
|  | 9.Gestural design | 5 | 5 | 4 | 4 | 3 | 4 |
|  | **Mean score** | 4.75 | 4.75 | 3.25 | 4.25 | 3.5 | 4.25 |
|  | 10.Layout | 5 | 5 | 4 | 5 | 3 | 3 |
|  | 11.Graphics | 5 | 4 | 5 | 4 | 4 | 4 |
|  | 12.Visual appeal | 5 | 4 | 5 | 3 | 5 | 3 |
|  | **Mean score** | 5.00 | 4.33 | 4.67 | 4.00 | 4.00 | 3.33 |
|  | 13.Accuracy of app description | 4 | 4 | 4 | 4 | 3 | 4 |
|  | 14.Goals | 5 | 4 | 5 | 4 | 4 | 3 |
|  | 15.Quality of information | 5 | 5 | 4 | 5 | 3 | 3 |
|  | 16.qantity of information | 5 | 5 | 3 | 5 | 3 | 3 |
|  | 17.Visual information | 5 | 5 | 4 | 4 | 4 | 3 |
|  | 18.Credibility | 4 | 5 | 4 | 5 | 4 | 4 |
|  | 19.Evidence base | n | n | n | n | n | n |
|  | **Mean score** | 4.67 | 4.67 | 4.00 | 4.50 | 3.50 | 3.33 |
|  | **Subtotal mean** | 4.85 | 4.49 | 4.08 | 3.94 | 3.70 | 3.33 |
|  | 20.recommend | 5 | 4 | 4 | 4 | 3 | 2 |
|  | 21.Use | 5 | 4 | 4 | 4 | 4 | 2 |
|  | 22.pay | 4 | 4 | 2 | 3 | 2 | 1 |
|  | 23.overall star rating | 5 | 4 | 2 | 3 | 3 | 2 |
|  | **Mean score** | 4.75 | 4.00 | 3.00 | 3.50 | 3.00 | 1.75 |
|  | 1.Awareness | 5 | 4 | 3 | 4 | 4 | 3 |
|  | 2.Knowledge | 5 | 4 | 3 | 5 | 4 | 4 |
|  | 3.Attitudes | 5 | 4 | 4 | 3 | 4 | 2 |
|  | 4.Intention to change | 5 | 4 | 4 | 3 | 2 | 2 |
|  | 5.Help seeking | 5 | 4 | 3 | 4 | 2 | 2 |
|  | 6.Behaviour change | 5 | 4 | 4 | 2 | 2 | 2 |
|  | **Mean score** | 5.00 | 4.00 | 3.50 | 3.50 | 3.00 | 2.50 |
| IMS | 1.Inform | 1 | 1 | 1 | 1 | 1 | 1 |
|  | 2.Instruct | 1 | 1 | 1 | 0 | 0 | 1 |
|  | 3.Record | 1 | 1 | 1 | 0 | 0 | 1 |
|  | 3.1 Collect data | 1 | 1 | 1 | 0 | 0 | 0 |
|  | 3.2 Share data | 1 | 1 | 1 | 0 | 0 | 0 |
|  | 3.3 Evaluate data | 1 | 0 | 0 | 0 | 0 | 0 |
|  | 3.4 Intervene | 1 | 0 | 1 | 0 | 0 | 0 |
|  | 4. Display | 1 | 1 | 1 | 0 | 0 | 0 |
|  | 5. Guide | 1 | 0 | 1 | 0 | 0 | 0 |
|  | 6. Remind of alert | 1 | 1 | 1 | 0 | 0 | 0 |
|  | 7. Communicate | 1 | 0 | 1 | 1 | 0 | 1 |
|  | Total | 11 | 7 | 10 | 2 | 1 | 3 |

IMS, IMS Institute for Healthcare Informatics functionality score; MARS, Mobile Application Rating Scale
